# Patient and healthcare practitioner evaluation of patient-reported outcomes in bariatric surgery – a modified Delphi study

**DOI:** 10.1038/s41366-024-01594-4

**Published:** 2024-07-26

**Authors:** Alyssa J. Budin, Priya Sumithran, Andrew D. MacCormick, Ian D. Caterson, Wendy A. Brown

**Affiliations:** 1 Department of Surgery, School of Translational Medicine, Monash University, The Alfred Centre, Melbourne, VIC Australia; 2 Department of Endocrinology and Diabetes, Alfred Health, Melbourne, VIC Australia; 3 Department of Surgery, The University of Auckland, Auckland, New Zealand; 4 Te Whatu Ora Counties Manukau, Otahuhu, Auckland New Zealand; 5 The Boden Initiative, Charles Perkins Centre, The University of Sydney, Camperdown, NSW Australia; 6 Department of Endocrinology, Royal Prince Alfred Hospital, Camperdown, NSW Australia; 7 Alfred Health, The Alfred Centre, Melbourne, VIC Australia

**Keywords:** Translational research, Public health

## Abstract

**Background:**

Patient-reported outcomes are an important emerging metric increasingly utilised in clinical, research and registry settings. These outcomes, while vital, are underutilised and require refinement for the specific patient population of those undergoing bariatric surgery. This study aimed to investigate and compare how pre-surgical patients, post-surgical patients, and healthcare practitioners evaluate patient-reported outcomes of bariatric surgery to identify outcomes that are considered most important.

**Methods:**

A modified Delphi survey was distributed to patients pre- and post-surgery, and to a variety of healthcare practitioners involved in bariatric care. Across two rounds, participants were asked to rate a variety of physical and psychosocial outcomes of bariatric surgery from 0 (Not Important) to 10 (Extremely Important). Outcomes rated 8–10 by at least 70% of participants were considered highly important (prioritised). The highest-rated outcomes were compared between the three groups as well as between medical and allied health practitioner subgroups.

**Results:**

20 pre-surgical patients, 95 post-surgical patients, and 28 healthcare practitioners completed both rounds of the questionnaire. There were 58 outcomes prioritised, with 21 outcomes (out of 90, 23.3%) prioritised by all three groups, 13 (14.4%) by two groups, and 24 (26.7%) prioritised by a single group or subgroup. Unanimously prioritised outcomes included ‘Co-morbidities’, ‘General Physical Health’, ‘Overall Quality of Life’ and ‘Overall Mental Health’. Discordant outcomes included ‘Fear of Weight Regain’, ‘Suicidal Thoughts’, ‘Addictive Behaviours’, and ‘Experience of Stigma or Discrimination’.

**Conclusion:**

While there was considerable agreement between stakeholder groups on many outcomes, there remain several outcomes with discordant importance valuations that must be considered. In particular, healthcare practitioners prioritised 20 outcomes that were not prioritised by patients, emphasising the range of priorities across stakeholder groups. Future work will consider these priorities to ensure resulting measures encompass all important outcomes and are beneficial and valid for end users.

## Introduction

Bariatric (metabolic) surgery is well-established as the most effective treatment currently available for obesity and its associated medical conditions. The rapidly increasing uptake of these procedures has generated a need for improved assessment and reporting of outcomes in this patient population. An important emerging metric is patient-reported outcomes which are increasingly employed in clinical, research and registry settings [1, 2]. A patient-reported outcome is “any report of the status of a patient’s health condition that comes directly from the patient” [3]. This may include descriptive, exploratory, or prognostic information on aspects of their quality of life, functional status, symptoms or symptom burden, associated medical conditions/co-morbidities, health behaviours, and experience of care. Such outcomes, while vital, are inconsistent and ill-defined in the bariatric field. Several reviews of bariatric and body contouring surgery have identified at least 68 validated measures, only some of which were validated in a bariatric population, and over 1000 different outcomes, the majority of which were reported in only a single paper [1, 2, 4]. This heterogeneity of reporting prevents meaningful interpretation of data and delays downstream implications on clinical practice.

Existing measures used to capture patient-reported outcomes in bariatric surgery are similarly limited, with the most commonly utilised patient-reported outcome measures (PROMs), the Beck Depression Inventory and the Medical Outcomes Short Form SF-36, being generic and non-specific to the experiences and outcomes of individuals undergoing bariatric surgery [1, 5, 6]. Bariatric-specific PROMs are limited in number, generally lacking content validity and failing to capture the patient perspective in their development [2, 6]. Despite their existence, and perhaps due to their inadequacy, PROMs are especially underutilised in the bariatric field. A study of Australian and Aotearoa New Zealand bariatric surgeons found that 61% of participants reported no collection of any patient-reported measure despite a general consensus that such data would be useful [5]. In the United States and England, studies have demonstrated that primary care physicians, general practitioners, and surgeons operating in various subspecialties find PROM data to be useful and impactful on clinical care, provided the PROM was specific to the condition or treatment being assessed and provided actionable data [7–11].

Given the heterogeneity of reporting, the lack of available and appropriate bariatric-specific PROMs, and the limited uptake of PROM collection, there is a need within the field to identify the most important outcomes to measure, and to choose or generate appropriate measures to capture these outcomes uniformly. Work has begun on identifying the outcomes and preferred PROMs to be used in research and clinical practice, including patient and healthcare practitioner stakeholders [2, 5, 12, 13]. While vital, this work is yet to be validated across settings and relies on the use of multiple existing PROMs which would be difficult to implement in large-scale data collections such as a Registry.

The Australian and New Zealand Bariatric Surgery Registry (ANZ BSR) is currently developing a PROM to incorporate patient outcomes in its data collection. This PROM will be concise, capturing only the most important outcomes to all stakeholders to allow for maximal data collection within the Registry population. This would enrich the quality of the Registry data set, allowing for ongoing quality and safety monitoring as well as research regarding the impacts of bariatric surgery on outcomes deemed most important by patients. Additionally, the concise Registry PROM has the potential to be implemented as a screening tool, following which clinicians and/or researchers may employ additional questionnaires to align with global standardisation projects. As a first step in this project, the current study aimed to identify the patient-reported outcomes considered the most important and impactful on an individual’s experience by surveying pre-surgical patients, post-surgical patients, and healthcare practitioners.

## Methods

### Generation of an item bank & questionnaire

Initial qualitative studies, including a scoping literature review, thematic analysis of online discussion boards, and focus groups, were undertaken by the ANZ BSR to provide an in-depth understanding of patients’ lived experiences. The outcomes and themes identified during these studies were used in a targeted literature search to identify commonly used validated PROMs in studies involving people living with obesity, people living with obesity undergoing bariatric surgery, and those awaiting bariatric surgery.

Items from identified PROMs were categorised and pooled by the domains and outcomes assessed. This formed the basis of a questionnaire developed to interrogate bariatric patients’ and healthcare practitioners’ opinions on the importance of the various outcomes. Where possible, similar concepts were combined to facilitate a shorter user-friendly survey. This resulted in an Outcome Importance Questionnaire containing 68 items across 10 domains; (1) General Health; (2) Eating Symptoms; (3) Sleep; (4) Sex; (5) Perception of Surgery; (6) General Quality of Life; (7) Social Activity; (8) Mental Health & Emotional Well-Being; (9) Eating Behaviour & Relationship to Food; and (10) Self-Esteem & Body Image.

### Outcome importance survey

The Outcome Importance Questionnaire was used to survey pre- and post-surgical bariatric patients, and a range of healthcare practitioners involved in the treatment and management of bariatric patients, including surgeons, nurses, dieticians, psychologists, and researchers.

Surgeons and healthcare practitioners based in Australia, actively contributing to the ANZ BSR were contacted via email, inviting them to complete the survey and to forward the survey to their colleagues and associated allied health practitioners. The survey was also distributed by the Australian and New Zealand Metabolic & Obesity Surgery Society (ANZMOSS) to target additional allied health practitioners.

Pre-surgical patients were recruited via participating healthcare practitioners, and post-surgical patients randomly selected from the ANZ BSR, who were over the age of 18 years and were not fully or partially opted out from the Registry, were invited to participate. These participants covered a range of demographics including age, sex, jurisdiction, public/private operations, diabetes status & type, surgery type, number of revisions, and time since surgery.

**Fig. 1:**
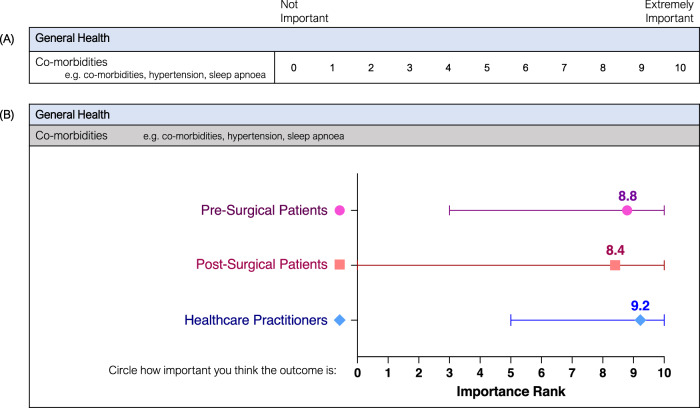
Example questionnaire items from the outcome importance questionnaire. (**A**) Round 1 questionnaire item 'Co-morbidities' in the General Health domain; (**B**) Round 2 questionnaire item 'Co-morbidities' in the General Health domain, displaying the range and mean importance scores from Round 1 for pre-surgical patients, post-surgical patients, and healthcare practitioners.

Participants completed the survey online via a secure online platform (Qualtrics, Provo, UT), or in paper form. Participants were asked to rate each outcome on a scale from 0 (not important) to 10 (extremely important). Participants were asked to consider whether an outcome has a significant impact on bariatric patients during their surgery and recovery and how important it is that this outcome be measured by doctors and researchers to better understand how surgery affects patients. Incorporating Delphi techniques, data generated from the Round 1 questionnaire were analysed and presented graphically within the Round 2 questionnaire, providing participants insight into the views of other stakeholder groups before asking them to rate the outcomes again. An example questionnaire item from Round 1 and Round 2 is presented in Fig. 1. Participants from Round 1 were sent 3 reminders to complete Round 2 via their nominated contact method before Round 2 was closed. In Round 2, participants were asked to rate each outcome from Round 1 again, as well as to rate additional outcomes that had been suggested by participants during Round 1. Participants were also asked to rank the 10 overall outcome domains from most to least important. This ranking question was included to elicit a forced response, combating a mild ceiling effect noted in Round 1. This provided insight into the order of importance across domains to further aid in refining the most important outcomes.

Full ethical approval for the study was obtained from the Alfred Hospital Ethics Committee (Project Number: 55/20).

### Analysis of questionnaire responses

Responses collected following Round 2 were organised and analysed by group. An outcome was identified as highly important (or prioritised) if at least 70% of participants in any group rated it an 8 or higher. As there are no standardised methods for the analysis of Delphi surveys, these criteria were modelled on similar studies within the field [13–15]. The number and percentage of prioritised outcomes for each group were calculated as well as the number and percentage of outcomes for which the groups overlapped. This was calculated for each individual item in the questionnaire as well as for each domain. Outcomes were then ranked based on the percentage of participants in each group who rated the outcome ≥8.

Healthcare practitioners were further sub-categorised as either a medical practitioner (physicians, surgeons), or an allied health practitioner (nurses, dieticians, psychologists, researchers). The number and percentage of prioritised outcomes were also determined for these subgroups.

The mean importance score was calculated for each domain by combining the scores for each outcome within a domain (Supplementary Table S1). Kruskal–Wallis tests and post hoc multiple comparisons with Bonferroni corrections were used to assess differences between groups for each questionnaire item. Differences in scores by demographic variables were assessed overall and within groups using the Kruskal–Wallis test for categorical variables, and Pearson correlations for continuous variables. The mean rank was calculated for each domain. Related-samples Friedman’s analysis of variance (ANOVA) and post hoc multiple comparisons with Bonferroni corrections were used to assess differences in ranked items.

Statistical analysis was performed using IBM SPSS Statistics (Version 27), with statistical significance inferred at a *p* value of <0.05.

## Results

A total of 313 participants took part in the study. The response rate for Round 2 was 45.7% (143 of 313) overall, with 41.0% (20 of 48), 52.8% (95 of 180), and 32.9% (28 of 85), of pre-surgical patients, post-surgical patients, and healthcare practitioners responding, respectively. The characteristics of the participants are presented in Table 1. Pre-surgical patients were younger than post-surgical patients (*p* < 0.001) and there was a higher proportion of women in the patient groups compared to the healthcare practitioner group (*p* < 0.05).

**Table 1:** Characteristics of pre-surgical patients, post-surgical patients, and healthcare practitioners participating in the study (*n* = 313).

Patients (*n* = 228)		
	Pre-surgical (*n* = 48)	Post-surgical (*n* = 180)
Mean age (SD)	42.3 (9.8)	49.2 (12.3)
Number female (%)	43 (89.6)	148 (82.2)
Ethnicity (%)		
Non-Indigenous Australian	38 (79.2)	141 (78.3)
Indigenous Australian or Torres Strait Islander	2 (4.2)	9 (78.3)
European	3 (6.3)	10 (5.6)
Other	5 (10.4)	20 (11.1)
Employment (%)		
Working full-time/self-employed	26 (54.2)	102 (56.7)
Working part-time/casual	13 (27.1)	36 (20.0)
Retired	–	13 (7.2)
Unable to work	3 (6.3)	5 (2.8)
Home duties	1 (2.1)	10 (5.6)
Student/apprentice	1 (2.1)	6 (3.3)
Unemployed	1 (2.1)	3 (1.7)
Others	3 (6.3)	5 (2.8)
Education (%)		
Less than Year 12 or equivalent	9 (18.8)	32(17.8)
Year 12 or equivalent	11 (22.9)	33 (18.3)
Trade/technical/vocational qualification	11 (22.9)	56 (31.1)
Undergraduate degree	11 (22.9)	30 (16.7)
Postgraduate degree	6 (12.5)	28 (15.6)
Bariatric procedure (%)^a^		
Adjustable gastric band (AGB)	–	39 (21.7)
Sleeve gastrectomy (SG)	41 (85.4)	108 (60.0)
Roux-en-Y gastric bypass (RYGB)	3 (6.3)	22 (12.2)
One anastomosis gastric bypass (OAGB)	2 (4.2)	7 (3.9)
Other	–	4 (2.2)
Not sure	2 (4.2)	–
Mean time since surgery (SD)	–	39.9 (53.4) months Range: 0–238

Healthcare practitioners (*n* = 85)		
Number female (%)	48 (56.5)	
*Prefer not to say*	1 (1.2)	
Profession (%)		
Bariatric surgeon	36 (42.4)	
Dietician	22 (25.8)	
Nurse specialist/nurse practitioner	15 (17.7)	
Psychologist/psychiatrist	7 (8.3)	
Bariatric physician/GP	3 (3.5)	
Researcher	2 (2.4)	
Years in profession (%)		
1–5 years	25 (29.4)	
6–10 years	18 (21.2)	
More than 10 years	42 (49.4)	
Ethnicity (%)		
Non-Indigenous Australian	58 (68.2)	
Indigenous Australian or Torres Strait Islander	-	
European	11 (12.9)	
Other	14 (16.5)	
Prefer not to say	2 (2.4)	

*SD* standard deviation.

^a^Planned procedure for pre-surgical patients; primary procedure for post-surgical patients.

17 participants (of 20; 85.0%) in the pre-surgical patient group underwent surgery in the time between the Round 1 and Round 2 questionnaires. They had a mean time since surgery of 6.53 months and 2 patients experienced complications of their procedure. There was no significant difference between patients in the pre-surgical group who had undergone surgery (converted pre-surgical patients) and those who had not, on any demographic variable. There was a significant difference in age between converted pre-surgical patients and post-surgical patients (*p* = 0.014). There was no significant within-group difference in results when converted patients were excluded from the analysis, however, significant differences were found between the existing groups on several outcomes. As such, participants remained in their original groups for analysis, however, the pre-surgical group may be considered as either pre-surgical or acutely post-surgical.

### Patient-reported outcome importance

Outcomes rated 8–10 by 70% of participants in at least two groups are presented in Table 2. All outcomes rated as highly important by any group or subgroup are available in Supplementary Table S2.

**Table 2:** Outcomes rated highly important (≥70% rating the outcome ≥8) by at least two groups (pre-surgical patients, post-surgical patients, healthcare practitioners).

Item	Pre-surgical patients (*n* = 46)	Post-surgical patients (*n* = 174)	Healthcare practitioners (*n* = 77)	Sig.
	% ≥8	% ≥8	% ≥8	
Outcomes rated highly important by all groups				
Co-morbidities *E.g. diabetes, hypertension, sleep apnoea*	85.0%	86.3%	100.0%	0.098
Overall quality of life, health and well-being	90.0%	87.4%	96.4%	0.211
Satisfaction with quality of life	85.0%	85.3%	96.4%	**0.026**
Satisfaction with surgery	85.0%	86.3%	92.9%	**0.043**
Weight/Surgery-specific symptoms *E.g. vomiting, regurgitation, heartburn, nausea, shortness of breath*	70.0%	73.7%	92.9%	**0.049**
Overall mental health	90.0%	80.0%	89.3%	**0.027**
General physical health *E.g. fitness, strength, endurance*	80.0%	89.5%	75.0%	0.062
Normality *(feeling able to live a “normal” life)*	80.0%	83.2%	89.3%	0.069
Outlook on life and expectations for the future	80.0%	83.2%	89.3%	0.986
Medication use	70.0%	72.6%	89.3%	0.163
Mobility *E.g. ability to walk, climb stairs, lift/carry groceries, bend or kneel*	80.0%	85.3%	85.7%	0.672
Self-esteem/Self-confidence	85.0%	81.1%	85.7%	0.278
Emotional eating	70.0%	70.5%	85.7%	0.858
Grazing/snacking behaviour	80.0%	72.6%	85.7%	0.110
Ability to care for oneself *E.g. dressing, bathing, grooming, or eating*	80.0%	84.2%	82.1%	0.345
Preparedness for bariatric surgery^a^ *(feeling informed and prepared for surgery, potential complications and outcomes)*	80.0%	70.5%	82.1%	0.439
Thoughts and feelings about physical self	80.0%	75.8%	75.0%	0.432
Relationship with spouse/partner or developing intimate relationships	80.0%	73.7%	71.4%	0.413
Eating patterns *(healthy and balanced eating patterns)*	75.0%	77.9%	78.6%	0.942
Confidence to engage in social activity	75.0%	76.8%	71.4%	0.176
Preoccupation with thoughts about body shape and/or size	75.0%	71.6%	71.4%	0.787
Outcomes rated highly important by pre- and post-surgical patients				
Feeling in control of weight and appearance	85.0%	74.7%	67.9%	0.317
Satisfaction with sleep	80.0%	75.8%	46.4%	**0.004**
Fear of returning habits^a^ *E.g. poor eating habits, activities, social habits*	75.0%	74.7%	60.7%	0.135
Fear of weight regain^a^	75.0%	70.5%	57.1%	0.126
Outcomes rated highly important by pre-surgical patients and healthcare practitioners				
Energy levels/fatigue	90.0%	60.0%	85.7%	**0.020**
Binge eating	75.0%	69.5%	89.3%	0.618
Ability to eat different types of food	85.0%	60.0%	71.4%	0.201
Physical signs *E.g. hair loss, teeth or gum problems, loss of sensation in hands and feet, skin irritations*	80.0%	42.1%	82.1%	**<0.001**
Feeling in control of eating behaviour^a^	80.0%	65.3%	71.4%	0.366
Depression	70.0%	68.4%	78.6%	0.134
Pain interference with day-to-day activities	70.0%	60.0%	78.6%	0.443
Impact of mental health on eating behaviour^a^	75.0%	62.1%	75.0%	0.247
Outcomes rated highly important by post-surgical patients and healthcare practitioners				
Level of pain	55.0%	89.5%	75.0%	**0.019**

Results are presented as percentage of participants within each group. Significance indicates differences between groups; pre-surgical patients, post-surgical patients and healthcare practitioners.

^a^Additional outcome suggested during Round 1 (received only one round of voting).

Bold values identify statistical significance (*p* < 0.05).

A total of 58 outcomes were prioritised by at least one group or subgroup. Pre-surgical patients, post-surgical patients, and healthcare practitioners rated 29, 26, and 44 outcomes as highly important, respectively. The highest-rated outcomes for each group were ‘Energy Levels/Fatigue’, ‘Overall Quality of Life’, and ‘Overall Mental Health’ (90.0% rated ≥8) for pre-surgical patients, ‘General Physical Health’ and ‘Level of Pain’ (89.5% rated ≥8) for post-surgical patients, and ‘Co-morbidities’ (100% rated ≥8) for healthcare practitioners.

There were 21 overlapping outcomes (out of 90, 23.3%) prioritised by all three groups and 18 outcomes (20.0%) classed as highly important by only a single group. Examples of unanimously prioritised outcomes include ‘Co-morbidities’, ‘Overall Quality of Life’, ‘Weight/Surgery-Specific Symptoms’, ‘Emotional Eating’, ‘Preparedness for Bariatric Surgery’, and ‘Self-Esteem’ (Table 2). Examples of discordant items prioritised by patients included ‘Level of Social Activity’, and ‘Quality of Sleep’ while discordant items prioritised by healthcare practitioners included ‘Suicidal Thoughts’, ‘Addictive Behaviours’, ‘Decision Remorse’, and ‘Experience of Stigma or Discrimination’ (Supplementary Table S2).

Pre-surgical patients prioritised 14 physical outcomes (of 32; 43.8%) and 23 psychosocial outcomes (of 58; 39.7%), post-surgical patients prioritised 8 physical outcomes (25.0%) and 18 psychosocial outcomes (31.0%) and healthcare practitioners prioritised 13 physical outcomes (40.6%) and 31 psychosocial outcomes (53.4%).

The percentage of medical and allied health practitioner sub-groups rating outcomes as highly important in Round 2 is presented in Supplementary Table S2. Of the 44 outcomes prioritised by healthcare practitioners, there were 28 overlapping outcomes between the subgroups, 5 prioritised by medical practitioners only and 11 prioritised by allied health practitioners only. There were an additional 3 outcomes rated as highly important by the allied health practitioner subgroup only and an additional 3 outcomes rated as highly important by the medical practitioner subgroup only (Supplementary Table S2). Medical practitioners prioritised 8 physical outcomes (25.0%) and 28 psychosocial outcomes (48.3%) while allied health practitioners prioritised 15 physical outcomes (46.9%) and 28 psychosocial outcomes (48.3%).

Mean combined scores for the overall domains for each group are presented in Table 3. ‘General Quality of Life’ had the highest mean rating across all three groups with the only significant difference between groups being for the domain ‘Perception of Surgery’. Mean domain scores for medical and allied health practitioner subgroups identified the highest-rated domains to be ‘General Quality of Life’ (Medical; 8.71 ± 0.75, Allied health; 8.89 ± 0.44), ‘Eating Behaviour & Relationship to Food’ (Medical; 8.31 ± 0.71, Allied health; 8.54 ± 0.78) and ‘General Health’ (Medical; 8.25 ± 1.00, Allied health; 8.20 ± 0.65). There were no significant differences between the subgroups in any domain.

**Table 3:** Round 2 average importance scores for pre-surgical patients, post-surgical patients, and healthcare practitioners for each domain.

Domain	Importance rating			
	Pre-surgical patients (*n* = 20)	Post-surgical patients (*n* = 94)	Healthcare practitioners (*n* = 28)	Sig.
General quality of life	9.06 (0.83)	8.72 (1.06)	8.81 (0.64)	0.378
Eating behaviour & relationship to food	8.20 (1.08)	7.98 (1.30)	8.41 (0.72)	0.249
General health	8.02 (0.84)	7.91 (0.78)	8.23 (0.80)	0.171
Sleep	8.11 (1.50)	7.83 (1.14)	7.52 (1.00)	0.236
Mental health & emotional well-being	7.81 (1.19)	7.55 (1.31)	8.06 (0.70)	0.158
Social well-being	8.02 (0.87)	7.78 (1.08)	7.90 (0.73)	0.609
Self-esteem & body image	7.89 (1.00)	7.76 (1.19)	7.83 (0.74)	0.884
Perception of surgery	7.72 (1.14)	6.98 (1.48)	7.62 (0.92)	**0.022**
Sex	7.68 (0.90)	7.24 (1.26)	7.23 (1.25)	0.367
Eating symptoms	7.25 (1.20)	6.96 (1.15)	7.37 (1.11)	0.208

Results are displayed as mean (SD).

Bold values identify statistical significance (*p* < 0.05).

In the post-surgical patient group, women had a higher overall score for the domains of ‘General Health’ (*p* = 0.024), ‘Eating Symptoms (*p* = 0.011), ‘Perception of Surgery’ (*p* = 0.010), ‘Social Well-Being’ (*p* = 0.004), ‘Mental Health & Emotional Well-Being’ (*p* = 0.017), ‘Eating Behaviour & Relationship to Food’ (*p* = 0.006), and ‘Self-Esteem & Body Image’ (*p* < 0.001). Female healthcare practitioners also rated the domain of ‘Eating Symptoms’ significantly higher than male healthcare practitioners (*p* = 0.030).

There was an inverse correlation between patient age and the importance score given to the domain of ‘Sex’ (r = −0.341, *p* = 0.001) by post-surgical patients (Supplementary Fig. S1). There were also inverse correlations between time since surgery and importance scores for the domain ‘Sleep’ (r = −0.192, *p* = 0.047) and ‘General Quality of Life’ (r = −0.280, *p* = 0.004) (Supplementary Fig. S2).

Finally, participants were asked to rank the overall domains from (1) most important to (10) least important. The results of the Friedman’s test indicated there was a statistically significant difference in ranks across the domains within each group, as well as overall (*p* < 0.001). The mean rank of each domain for each group is listed in Table 4.

**Table 4:** Mean rank and rank order of domains for all participants, pre-surgical patients, post-surgical patients, and healthcare practitioners.

Domain	Total (*n* = 123)		Pre-surgical patients (*n* = 17)		Post-surgical patients (*n* = 80)		Healthcare practitioners (*n* = 26)		
	Mean rank	Rank order	Mean rank	Rank order	Mean rank	Rank order	Mean rank	Rank order	Sig.
General health	2.54	1	2.68	1	2.88	1	1.42	1	**0.027**
General quality of life	3.29	2	4.15	2	3.26	2	2.81	2	0.075
Mental health & emotional well-being	4.77	3	4.85	4	4.99	3	4.04	3	0.249
Eating symptoms	4.96	4	4.38	3	5.19	5	4.65	4	0.463
Eating behaviour & relationship with food	5.25	5	5.09	5	5.13	4	5.73	5	0.597
Self-esteem & body-image	5.77	6	5.74	6	5.54	6	6.5	6	0.323
Sleep	6.66	7	5.79	7	6.71	8	7.08	8	0.344
Sex	6.99	8	7.62	10	6.51	7	8.04	10	**0.009**
Social well-being	7.13	9	7.5	9	7.16	9	6.81	7	0.39
Perception of surgery	7.63	10	7.21	8	7.63	10	7.92	9	0.742

Results are presented as mean rank and rank order within each group. Significance indicates differences in mean rank between groups.

Bold values identify statistical significance (*p* < 0.05).

## Discussion

This study identified and compared the patient-reported outcomes of bariatric surgery considered the most important and impactful on an individual’s experience by pre-surgical patients, post-surgical patients, and healthcare practitioners. Following two rounds of voting, 21 outcomes were unanimously prioritised by all three groups with an additional 37 outcomes prioritised by at least one group. The highest-rated outcomes across the groups were ‘Co-morbidities’, ‘General Physical Health’, ‘Energy Levels / Fatigue’, ‘Level of Pain’, ‘Overall Quality of Life’, and ‘Overall Mental Health’. The most important domains across the study were ‘General Quality of Life’, ‘General Health’, and ‘Mental Health & Emotional Well-Being’.

Several other studies have investigated the importance valuations of bariatric surgery outcomes. This includes a global study by de Vries et al. which identified the most important domains to be ‘Self-Esteem’, ‘Physical Health’ and ‘Mental / Psychological Health’ [13]. A study by Coulman et al. surveyed post-surgical patients and healthcare practitioners in the UK with endorsed items including ‘improvements in diabetes’, ‘improvement in obstructive sleep apnoea symptoms’, ‘being able to accomplish work tasks’, ‘being able to stop eating when feeling full’, ‘normality’, and ‘having a positive outlook on life and expectations for the future’ [15]. A similar study conducted in the USA by Greene et al. used focus groups to identify the most important outcomes according to healthcare practitioners and the most important domains according to pre- and post-surgical patients [16]. They identified the most important domains across groups to be ‘Health’, ‘Self-Confidence’, and ‘Mobility’, aligning with our domains ‘General Health’, and ‘Self-Esteem & Body Image’. While these studies provide evidence that priorities are similar across countries, our study included a more comprehensive list of outcomes, particularly in the psychosocial well-being domains. This, alongside sufficient recruitment numbers to compare between medical and allied health practitioners, allowed for the quantitative comparison of individual items within and across domains for multiple stakeholder groups. This reveals the distinct priorities of pre- and post-surgical patients, and the variety of healthcare practitioners included in our study. This is crucial for the development of a concise PROM for a Registry setting as the PROM must collect the most important information with the fewest items.

The contrasts between patient and healthcare practitioner importance valuations have been widely investigated and highlighted across multiple disease states. In general, the literature suggests that patients tend to rate psychosocial outcomes such as quality of life, mental health, and emotional well-being higher than healthcare practitioners, while practitioners tend to favour clinical outcomes such as the resolution of comorbidities and physical functioning [13, 15, 17–21]. In contrast, healthcare practitioners in our study prioritised a higher percentage of psychosocial outcomes (53.4%) compared to pre-surgical and post-surgical patient groups (39.7% and 31.0%, respectively). This observation was consistent when dividing healthcare practitioners into medical and allied health practitioner subgroups. Despite evidence suggesting that allied health practitioners are more likely to prioritise psychosocial outcomes compared to medical practitioners [15], this was not found in our results with medical and allied health practitioner groups each prioritising 28 psychosocial outcomes (48.3%). This may reflect the emphasis in our study on patient-reported outcomes to be collected in conjunction with traditional clinical outcomes, allowing healthcare practitioners to focus on additional outcomes that would be most impactful on their patients. These results may also be indicative of the developing recognition of both psychosocial well-being and patient-reported outcomes as important in the bariatric field, particularly given the inherent risk of adverse outcomes in this patient cohort [22–24]. The COVID-19 pandemic has also brought mental health and psychosocial well-being to the forefront of clinical considerations, particularly in patients living with obesity and those undergoing bariatric procedures during this time [25–27]. The combination of these factors in our results may indicate a much-needed shift in perspective from healthcare practitioners beginning to evaluate these outcomes as highly important.

Although there were some differences in the number and type of outcomes prioritised between groups, there was a good level of agreement on the mean importance scores and ranks for each domain. The only significant difference following Round 2 was for the domain of ‘Perception of Surgery’ in which pre-surgical patients and healthcare practitioners rated it significantly higher than post-surgical patients. This difference highlights the importance healthcare practitioners place on the patients’ experience of surgery, which may be considered a direct reflection of the surgeon’s performance. In the patient groups, pre-surgical patients may feel a level of trepidation or anxiety in the lead-up to surgery and subsequently place a high level of importance on this domain. Conversely, post-surgical patients (some of whom were up to 21 years post-surgery) are no longer focused on the procedure itself, and place more importance on the outcomes affecting their day-to-day life.

The differences between pre- and post-surgical patients are further emphasised when considering their priorities relative to their surgery. With a time since surgery ranging from 0 to 256 months (21 years), opinions have been captured from patients acutely post-surgery through to patients who are well past the ‘honeymoon’ phase of the 1^st^ year postoperatively. This range of experiences enriches the data set and allows for a better interpretation of how outcomes may be more impactful at various time points. The correlations between time since surgery and both the ‘Sleep’ and ‘General Quality of Life’ domains highlight how patient priorities shift relative to their procedure, while the correlation between the domain of ‘Sex’ and participant age emphasises how priorities can vary depending on stage of life. Additional research is needed to identify how, why, and when these priorities change and whether this is an impact of bariatric surgery or simply a change in perspective as people age. These differences highlight the potential for patient subgroups to place a higher value on certain outcomes compared to the consensus. As the work continues, it will be important to identify any subgroups that may experience distinct outcomes affecting their health and well-being post-surgery, including patients from diverse ethnic, gender, and socioeconomic backgrounds. This could prove important in identifying patients experiencing poor outcomes post-surgery to provide enhanced, and individualised patient care.

This study is strengthened by the breadth of experiences collected from both pre- and post-surgical patients where previous studies have included only post-surgical patients [15], as well as the inclusion of a variety of healthcare practitioners, with sufficient numbers to compare medical and allied health subgroups. The response rates of 41.0%, 52.8%, and 32.9% for pre-surgical patients, post-surgical patients, and healthcare practitioners, respectively is a limitation of this study. The results may not be indicative of the whole population of both patients and healthcare practitioners across Australia. However, attrition is typical for Delphi studies, with current response rates higher than similar studies in the field [15, 28]. In addition, a sample size between 10 to 50 is generally considered optimal for Delphi studies although no standardised sample size exists and published studies can range from 10 to 1000 participants [29–31]. The demographics of our participants were also generally representative of the population of interest. Patients were predominantly female (89.6% pre-surgical and 82.2% post-surgical), of non-indigenous Australian ethnicity (79.2% and 78.3%), with a mean age of 42.3 years for pre-surgical patients and 49.2 for post-surgical patients. This is in line with the population of individuals undergoing bariatric surgery in Australia, with the ANZ BSR reporting the majority of participants to be female (79.7%) with a mean age of 41.4 years at time of operation [32].

Although the sample is generally representative, results are still limited in ethnic and gender minority groups who may experience bariatric surgery differently. Sex differences in 8 of 10 domains for post-surgical patients in this study may represent interesting potential differences in patient experiences post-surgery. However, with the generally lower participation of men in both bariatric surgery and subsequent research, these results should be considered cautiously. It cannot be determined whether sex differences identified in this study are due to real-world differences in outcomes, or a tendency for women to rate outcomes higher than men overall. Future work may focus on ethnic and gender minority groups, particularly male and gender-diverse individuals, as well as indigenous Aboriginal and Torres Strait Islander peoples in Australia and Māori in Aotearoa New Zealand, to better understand how outcomes may differ in these patient populations.

This study incorporates the views of multiple stakeholder groups in understanding which outcomes of bariatric surgery are the most important and impactful. These results will be utilised in the development of a patient-reported outcome measure (PROM) specifically for the ANZ BSR. By developing a concise PROM for a Registry setting we aim to facilitate widespread data collection with the potential to enable ongoing quality and safety monitoring, additional research efforts, as well as providing a minimum dataset that can be expanded to include outcomes and measures aligned with global standardisation projects. The 58 prioritised outcomes will be taken to focus groups and consultations with both patients and healthcare practitioners to generate a pilot PROM. It is expected that the unanimously prioritised outcomes, as well as the highest-rated outcomes for each stakeholder group will be included in the pilot PROM. In line with the aims of other studies, the standardisation of patient-reported data at a Registry level will facilitate large-scale data collection, improving the collective quality of the data while avoiding implementation barriers faced by individual surgeons and hospitals [5]. Future work should focus on aligning outcomes across clinical settings, research, and registries nationally and internationally to allow for a better understanding of the bariatric surgery landscape, including identifying those patients who may be at risk for developing adverse outcomes.

## Conclusion

This research identified those outcomes considered most important by pre-surgical patient, post-surgical patient, and healthcare practitioner groups. Of importance was highlighting the differences between patients and healthcare practitioners, as well as between pre- and post-surgical groups, and medical & allied health practitioners. This research emphasises the range of priorities represented across these groups and the need for any core outcome set or PROM to consider these priorities.

## Supplementary information

Supplementary Material

## Data Availability

The datasets generated during and/or analysed during the current study are available from the corresponding author on reasonable request.
