# Supplementary material for: Patient and healthcare practitioner evaluation of patient-reported outcomes in bariatric surgery – a modified Delphi study
Source: Int J Obes (Lond). 2024 Jul 26;48(10):1489–97. doi: 10.1038/s41366-024-01594-4 (PMC11420076; doi:10.1038/s41366-024-01594-4)
Supplement: Supplementary file 1 — Supplementary Material [file 41366_2024_1594_MOESM1_ESM.docx]

# Patient and Healthcare Practitioner Evaluation of Patient-Reported Outcomes in Bariatric Surgery – A Modified Delphi Study

Ms Alyssa J. Budin, Associate Professor Priya Sumithran, Associate Professor Andrew D. MacCormick, Professor Ian Caterson, & Professor Wendy A. Brown.

# Supplementary Material

Table S1. The outcomes and domains identified during the development of the Item Bank and Outcome Importance Questionnaire

Table S2. Items rated highly important (≥ 70% rating the item ≥ 8) by Pre-surgical Patients, Post-Surgical Patients, and Healthcare Practitioners including Medical and Allied Health Practitioner subgroups

**Figure S1.** Correlation between mean importance score for the domain ‘Sex’ and participant age in the post-surgical patient group

**Figure S2:** Correlation between post-surgical patient time since surgery (months) and mean importance score for the domains (A) ‘General Quality of Life’ and (B) ‘Sleep’

Table S1. The outcomes and domains identified during the development of the Item Bank and Outcome Importance Questionnaire

| **Domains** | **Outcomes** |
| --- | --- |
| **General Health** | Co-morbidities |
|  | Medication use |
|  | Physical Signs |
|  | Non-specific symptoms |
|  | Digestive Symptoms |
|  | Weight / Surgery-specific symptoms |
|  | General physical health |
|  | Mobility |
|  | Ability to care for oneself |
|  | Energy levels / Fatigue |
|  | Level of pain |
|  | Pain interference with day-to-day activities |
|  | Development of excess skin or skin folds |
|  | Fertility |
| **Eating Symptoms** | Ability to eat different types of food |
|  | Amount of food that can be eaten in one sitting |
|  | Ability to have food and drink together |
|  | Tolerance for alcohol |
|  | Change in appetite |
|  | Ability to tolerate fluids |
|  | Variation in food/fluid tolerance |
|  | Maladaptive eating habits |
| **Sleep** | Ability to fall asleep |
|  | Quality of sleep |
|  | Satisfaction with sleep |
|  | Snoring |
| **Sex** | Physical ability to engage in sexual activity |
|  | Interest in sexual activity |
|  | Enjoyment / pleasure from sexual activity |
|  | Satisfaction with sex life |
|  | Hormonal changes |
|  | Loss or change in libido / impotence |
| **Perception of Surgery** | Satisfaction with surgery |
|  | Decision remorse |
|  | Feeling uncomfortable or conscious of surgical outcomes |
|  | Fear of surgical outcomes affecting others |
|  | Desire for ‘cosmetic’ procedures |
|  | Unmet expectations |
|  | Preparedness for bariatric surgery |
|  | Surgery secrecy |
| **General Quality of Life** | Overall quality of life, health and well-being |
|  | Satisfaction with quality of life |
|  | Normality |
|  | Outlook on life and expectations for the future |
| **Social Activity** | Level of social activity |
|  | Confidence to engage in social activity |
|  | Relationship with spouse/partner or developing intimate relationships |
|  | Relationships with family members |
|  | Relationships with friends |
|  | Support network |
|  | Feelings of social isolation |
|  | Experience of stigma or discrimination |
|  | Comfort engaging in social eating situations |
|  | Impact of physical outcomes on social life |
| **Mental Health & Emotional Well-Being** | Overall mental health |
|  | Mood swings |
|  | Depression |
|  | Anhedonia |
|  | Self-harm behaviours or thoughts |
|  | Suicidal thoughts |
|  | Anxiety |
|  | Fear of negative evaluation |
|  | Feeling in control of thoughts and feelings |
|  | Impulsivity |
|  | Irritability |
|  | Coping |
|  | Self-efficacy |
|  | Cognitive function |
|  | Addictive behaviours |
|  | Feelings of guilt |
|  | Fear of weight regain |
|  | Fear of returning habits |
| **Eating Behaviour & Relationship to Food** | Binge Eating |
|  | Emotional Eating |
|  | Grazing / Snacking behaviour |
|  | Use of diet pills, laxatives, or intentional vomiting to control weight |
|  | Eating patterns |
|  | Preoccupation with thoughts of food |
|  | Feeling guilty or disappointed after eating |
|  | Feelings of pleasure from eating |
|  | Feeling in control of eating behaviour |
|  | Impact of mental health on eating behaviour |
| **Self-Esteem & Body Image** | Self-esteem / Self-confidence |
|  | Thoughts and feelings about physical self |
|  | Preoccupation with thoughts about body shape and/or size |
|  | Thoughts and feelings about excess skin or skin folds |
|  | Feeling in control of weight and appearance |
|  | Avoidance of situations, people or activities because of body image |
|  | Body dysmorphia |
|  | Comparison to others |

Table S2. Items rated highly important (≥ 70% rating the item ≥ 8) by Pre-surgical Patients, Post-Surgical Patients, and Healthcare Practitioners including Medical and Allied Health Practitioner subgroups

| **Item** | **Pre-Surgical Patients**  (n = 39) | | **Post-Surgical Patients**  (n = 39) | | **Healthcare Practitioners**  (n = 39) | | **Sig.** ^(a)^ | **Medical Practitioners**  (n = 39) | | **Allied Health Practitioners***  (n = 39) | | **Sig.** ^(b)^ |
| --- | --- | --- | --- | --- | --- | --- | --- | --- | --- | --- | --- | --- |
|  | Md  (25%-75%) | % ≥ 8 | Md  (25%-75%) | % ≥ 8 | Md  (25%-75%) | % ≥ 8 |  | Md  (25%-75%) | % ≥ 8 | Md  (25%-75%) | % ≥ 8 |  |
| **Items rated highly important by all groups** | | | | | | | | | | | | |
| Co-morbidities  *E.g. diabetes, hypertension, sleep apnoea* | 9 (8 - 9.75) | 85.0% | 9 (8 - 9) | 86.3% | 9 (9 - 10) | 100.0% | 0.098 | 9 (9 - 10) | 100.0% | 9 (8 - 9) | 100.0% | **0.049** |
| Overall quality of life, health and well-being | 10 (9 - 10) | 90.0% | 9 (8 - 10) | 87.4% | 9 (8 - 10) | 96.4% | 0.211 | 9 (8 - 10) | 100.0% | 9 (9 - 9.5) | 92.9% | 0.827 |
| Satisfaction with quality of life | 10 (9 - 10) | 85.0% | 9 (8 - 10) | 85.3% | 9 (8 - 10) | 96.4% | **0.026** | 9 (8 - 10) | 100.0% | 9 (9 - 10) | 92.9% | 0.340 |
| Satisfaction with surgery | 10 (9 - 10) | 85.0% | 9 (8 - 10) | 86.3% | 9 (8 - 9) | 92.9% | **0.043** | 9 (8 - 9) | 100.0% | 9 (8 - 9) | 85.7% | 0.715 |
| Weight / Surgery-specific symptoms  *E.g. vomiting, regurgitation, heartburn, nausea, shortness of breath* | 8 (7 - 9) | 70.0% | 8 (7 - 9) | 73.7% | 9 (8 - 10) | 92.9% | **0.049** | 9 (8 - 10) | 92.3% | 9 (8 - 9.25) | 92.9% | 0.445 |
| Overall mental health | 9.5 (8 - 10) | 90.0% | 9 (8 - 9) | 80.0% | 9 (8 - 10) | 89.3% | **0.027** | 9 (8 - 10) | 92.3% | 9 (9 - 9.5) | 85.7% | 0.871 |
| General Physical Health  *E.g. fitness, strength, endurance* | 9 (8 - 9) | 80.0% | 9 (8 - 9) | 89.5% | 8 (7.25 - 9) | 75.0% | 0.062 | 9 (8 - 9.5) | 84.6% | 8 (7 - 8) | 64.3% | **0.005** |
| Normality  *(feeling able to live a "normal" life)* | 10 (8 - 10) | 80.0% | 9 (8 - 10) | 83.2% | 8 (8 - 9) | 89.3% | 0.069 | 8 (8 - 9) | 84.6% | 8 (8 - 9) | 92.9% | 0.552 |
| Outlook on life and expectations for the future | 9 (8 - 10) | 80.0% | 9 (8 - 9) | 83.2% | 9 (8 - 9) | 89.3% | 0.986 | 8 (8 - 9) | 92.3% | 9 (8 - 9) | 85.7% | 0.281 |
| Medication Use | 8.5 (7 - 9.75) | 70.0% | 8 (7.25 - 9) | 72.6% | 8 (8 - 10) | 89.3% | 0.163 | 9 (8 - 10) | 92.3% | 8 (8 - 9) | 92.9% | 0.141 |
| Mobility  *E.g. ability to walk, climb stairs, lift/carry groceries, bend or kneel* | 9 (8 - 9.75) | 80.0% | 9 (8 - 9) | 85.3% | 8 (8 - 9) | 85.7% | 0.672 | 9 (8 - 9.5) | 84.6% | 8 (8 - 8.25) | 85.7% | 0.111 |
| Self-esteem / Self-confidence | 9 (8 - 9.25) | 85.0% | 9 (8 - 9) | 81.1% | 8 (8 - 9) | 85.7% | 0.278 | 8 (8 - 9) | 84.6% | 8 (8 - 9) | 85.7% | 0.782 |
| Emotional Eating | 9 (7.5 - 10) | 70.0% | 9 (7 - 10) | 70.5% | 9 (8 - 9) | 85.7% | 0.858 | 8 (8 - 9) | 84.6% | 9 (8 - 10) | 92.9% | 0.159 |
| Grazing / Snacking behaviour | 9 (8.5 - 10) | 80.0% | 8 (8 - 10) | 72.6% | 9 (8 - 9.25) | 85.7% | 0.11 | 9 (8 - 9.75) | 84.6% | 9 (8 - 9.5) | 92.9% | 0.625 |
| Ability to care for oneself  *E.g. dressing, bathing, grooming, or eating* | 9 (8 - 10) | 80.0% | 9 (8 - 9) | 84.2% | 8 (8 - 9.75) | 82.1% | 0.345 | 8 (8 - 10) | 84.6% | 8 (7.75 - 9) | 78.6% | 0.496 |
| *^#^* Preparedness for bariatric surgery  *(feeling informed and prepared for surgery, potential complications and outcomes)* | 9 (8 - 10) | 80.0% | 8 (7 - 9.5) | 70.5% | 9 (8 - 10) | 82.1% | 0.439 | 8 (5.75 - 9) | 76.9% | 9 (8 - 10) | 85.7% | 0.336 |
| Thoughts and feelings about physical self | 8 (8 - 9.25) | 80.0% | 8 (8 - 9) | 75.8% | 8 (8 - 9) | 75.0% | 0.432 | 8 (7.25 - 9) | 69.2% | 8 (8 - 8) | 78.6% | 0.740 |
| Relationship with spouse/partner or developing intimate relationships | 9 (8 - 10) | 80.0% | 8 (8 - 9) | 73.7% | 8 (7.75 - 9) | 71.4% | 0.413 | 9 (8 - 9) | 84.6% | 8 (7 - 8) | 57.1% | **0.016** |
| Eating patterns  *(healthy and balanced eating patterns)* | 9 (8 - 9.5) | 75.0% | 9 (8 - 9.75) | 77.9% | 8 (8 - 9.25) | 78.6% | 0.942 | 8.5 (8 - 9) | 76.9% | 9 (8 - 10) | 85.7% | 0.514 |
| Confidence to engage in social activity | 9 (8 - 9) | 75.0% | 8 (8 - 9) | 76.8% | 8 (7.75 - 8.25) | 71.4% | 0.176 | 8 (8 - 8.75) | 76.9% | 8 (7 - 8.5) | 64.3% | 0.533 |
| Preoccupation with thoughts about body shape and/or size | 8 (7.75 - 9) | 75.0% | 8 (7.75 - 9) | 71.6% | 8 (7.75 - 9) | 71.4% | 0.787 | 8 (7.25 - 9) | 69.2% | 8 (7.5 - 8.5) | 71.4% | 0.861 |
| **Items rated highly important by pre- and post-surgical patients** | | | | | | | | | | | | |
| Feeling in control of weight and appearance | 8 (8 - 9.25) | 85.0% | 8 (8 - 9) | 74.7% | 8 (7 - 9) | 67.9% | 0.317 | 8 (7 - 8.75) | 69.2% | 8 (7 - 8.5) | 64.3% | 0.850 |
| Satisfaction with sleep | 8 (8 - 10) | 80.0% | 8 (8 - 9) | 75.8% | 7 (7 - 8) | 46.4% | **0.004** | 7 (7 - 8.75) | 38.5% | 7.5 (7 - 8) | 50.0% | 0.979 |
| *^#^* Fear of returning habits  *e.g. poor eating habits, activities, social habits* | 9 (8 - 10) | 75.0% | 9 (8 - 10) | 74.7% | 8 (7 - 9) | 60.7% | 0.135 | 8 (7 - 9) | 61.5% | 8 (7 - 8) | 57.1% | 0.628 |
| *^#^* Fear of weight regain | 9 (8 - 10) | 75.0% | 9 (7 - 10) | 70.5% | 8 (7 - 9) | 57.1% | 0.126 | 8 (7 - 9) | 61.5% | 8 (7 - 8) | 50.0% | 0.381 |
| **Items rated highly important by pre-surgical patients and healthcare practitioners** | | | | | | | | | | | | |
| Energy levels / Fatigue | 9 (8 - 9.75) | 90.0% | 8 (7 - 9) | 60.0% | 8 (8 - 9) | 85.7% | **0.02** | 8 (7.5 - 9) | 76.9% | 8 (8 - 8.25) | 92.9% | 0.914 |
| Binge Eating | 9 (8 - 9) | 75.0% | 8 (7 - 9) | 69.5% | 9 (8 - 9.25) | 89.3% | 0.618 | 8 (8 - 9) | 84.6% | 9 (8 - 9.5) | 92.9% | 0.430 |
| Ability to eat different types of food | 8 (8 - 9.75) | 85.0% | 8 (7 - 9) | 60.0% | 8 (7 - 8.75) | 71.4% | 0.201 | 8 (7 - 8.5) | 61.5% | 8 (7.75 - 9) | 78.6% | 0.410 |
| Physical signs  *E.g. hair loss, teeth or gum problems, loss of sensation in hands and feet, skin irritations* | 8 (8 - 9) | 80.0% | 7 (6.25 - 8) | 42.1% | 8 (8 - 9) | 82.1% | **<0.001** | 8 (7 - 9) | 61.5% | 8 (8 - 9) | 100.0% | 0.100 |
| *^#^* Feeling in control of eating behaviour | 8 (8 - 10) | 80.0% | 8 (7 - 9) | 65.3% | 8 (7.75 - 9) | 71.4% | 0.366 | 8 (7 - 9) | 61.5% | 8 (8 - 9) | 78.6% | 0.512 |
| Depression | 8 (7 - 10) | 70.0% | 8 (7 - 9) | 68.4% | 9 (8 - 9) | 78.6% | 0.134 | 9 (8 - 9) | 84.6% | 8 (7.5 - 9) | 71.4% | 0.258 |
| Pain interference with day-to-day activities | 8 (7 - 9) | 70.0% | 8 (7 - 9) | 60.0% | 8 (8 - 9) | 78.6% | 0.443 | 8 (7 - 9) | 69.2% | 8 (8 - 8.25) | 85.7% | 0.772 |
| *^#^* Impact of mental health on eating behaviour | 9 (8 - 9) | 75.0% | 8 (7 - 9) | 62.1% | 8 (8 - 9) | 75.0% | 0.247 | 8 (7 - 9) | 61.5% | 8 (8 - 9) | 85.7% | 0.326 |
| **Items rated highly important by post-surgical patients & healthcare practitioners** | | | | | | | | | | | | |
| Level of pain | 8 (7 - 9) | 55.0% | 9 (8 - 9) | 89.5% | 8 (7.25 - 9) | 75.0% | **0.019** | 8 (7.5 - 9) | 76.9% | 8 (7 - 8.25) | 71.4% | 0.560 |
| **Items rated highly important by pre-surgical patients only** | | | | | | | | | | | | |
| Level of social activity | 8 (8 - 9) | 75.0% | 8 (7.5 - 9) | 69.5% | 8 (7 - 8.25) | 67.9% | 0.406 | 8 (7.25 - 9) | 69.2% | 8 (7 - 8) | 64.3% | 0.448 |
| *^#^* Ability to tolerate fluids | 8 (7 - 9.75) | 70.0% | 8 (6 - 9) | 50.5% | 8 (7 - 10) | 67.9% | **0.021** | 8 (6.5 - 10) | 61.5% | 8 (7 - 8) | 71.4% | 0.940 |
| *^#^* Variation in food/fluid tolerance  *E.g. what can or cannot be eaten is unpredictable or changes from day to day* | 8 (7 - 8.75) | 70.0% | 7 (6 - 8.25) | 42.1% | 8 (7 - 9) | 57.1% | 0.129 | 8 (5.5 - 9) | 53.8% | 8 (7 - 8) | 57.1% | 0.505 |
| Quality of sleep | 8.5 (7 - 10) | 70.0% | 8 (7 - 9) | 65.3% | 8 (7 - 8) | 53.6% | 0.108 | 8 (7 - 8.75) | 53.8% | 7.5 (7 - 8) | 50.0% | 0.512 |
| **Items rated highly important by healthcare practitioners only** | | | | | | | | | | | | |
| Suicidal thoughts | 7 (5.75 - 10) | 40.0% | 8 (6 - 9) | 53.7% | 9 (8 - 9.25) | 89.3% | **0.005** | 9 (8 - 9.75) | 92.3% | 9 (8 - 9) | 85.7% | 0.772 |
| Addictive behaviours  *E.g. alcohol, drug use, gambling* | 8 (5 - 9) | 55.0% | 8 (6 - 9) | 50.5% | 8 (8 - 9) | 85.7% | **0.035** | 8 (8 - 9) | 84.6% | 8 (8 - 9) | 85.7% | 0.763 |
| Feelings of pleasure from eating | 8 (6 - 8.5) | 60.0% | 8 (7 - 9) | 50.5% | 8 (8 - 9) | 82.1% | 0.228 | 8 (8 - 9) | 84.6% | 8 (8 - 9) | 78.6% | 0.835 |
| Decision remorse  *(feeling of anxiety or regret about the decision to undergo surgery)* | 9 (7 - 9) | 60.0% | 7 (5 - 9) | 45.3% | 9 (8 - 10) | 82.1% | **0.001** | 9 (8 - 10) | 84.6% | 9 (8 - 9) | 78.6% | 0.505 |
| Self-harm behaviours or thoughts | 8 (5.75 - 10) | 55.0% | 8 (6.75 - 9) | 56.8% | 9 (8 - 9) | 82.1% | **0.003** | 9 (8 - 9.75) | 84.6% | 9 (8 - 9) | 78.6% | 0.624 |
| Anxiety | 8 (7 - 9.25) | 65.0% | 8 (7 - 9) | 60.0% | 8 (8 - 9) | 82.1% | 0.377 | 8 (8 - 9) | 76.9% | 8 (8 - 9) | 85.7% | 0.856 |
| Experience of stigma or discrimination | 8 (6 - 9) | 60.0% | 8 (6 - 9) | 48.4% | 8 (8 - 9) | 82.1% | **0.01** | 9 (8 - 9.75) | 84.6% | 8 (8 - 8.5) | 78.6% | 0.111 |
| Feeling guilty or disappointed after eating | 8 (7 - 10) | 60.0% | 8 (7 - 9) | 52.6% | 8 (8 - 9) | 75.0% | 0.568 | 8 (8 - 8.75) | 76.9% | 8 (7.5 - 9) | 71.4% | 0.437 |
| Preoccupation with thoughts of food | 8 (7 - 9.5) | 65.0% | 8 (7 - 9) | 61.1% | 8 (7.75 - 9) | 71.4% | 0.912 | 8 (8 - 8.75) | 76.9% | 8 (7 - 9) | 64.3% | 0.908 |
| *^#^* Unmet expectations  *(feelings of failure or not meeting expected surgical outcomes)* | 8 (6 - 8) | 55.0% | 7 (6 - 9) | 44.2% | 8 (7 - 9) | 71.4% | 0.123 | 8 (7 - 9) | 69.2% | 8 (7.5 - 8.5) | 71.4% | 0.936 |
| *^#^* Maladaptive eating habits  *E.g. findings ways to “cheat” surgery, many small meals, calorie-dense foods, liquid calories* | 7.5 (6 - 8.75) | 50.0% | 8 (6 - 9) | 51.6% | 9 (7 - 10) | 71.4% | **0.017** | 8 (7 - 9.5) | 69.2% | 9 (7 - 9) | 71.4% | 0.330 |
| *^#^* Fertility | 6.5 (3.5 - 9) | 45.0% | 6 (1.75 - 8) | 34.7% | 8 (7 - 10) | 71.4% | **<0.001** | 8 (6.5 - 9.5) | 61.5% | 8 (7.75 - 10) | 78.6% | 0.317 |
| Feelings of social isolation | 7 (7 - 8) | 45.0% | 7 (7 - 9) | 44.2% | 8 (7.75 - 9) | 71.4% | 0.081 | 8 (8 - 9) | 76.9% | 8 (7 - 8.5) | 64.3% | 0.353 |
| Use of diet pills, laxatives, or intentional vomiting to control weight | 7 (6 - 9) | 45.0% | 8 (6 - 9) | 50.5% | 9 (7.75 - 10) | 71.4% | **0.036** | 9 (7.25 - 10) | 69.2% | 9 (7.5 - 9.5) | 71.4% | 0.895 |
| **Items rated highly important by Medical practitioners only** | | | | | | | | | | | | |
| Relationships with friends | 8 (7 - 9) | 60.0% | 8 (7 - 9) | 57.9% | 8 (7 - 8) | 53.6% | 0.729 | 8 (8 - 8) | 76.9% | 7 (7 - 8) | 28.6% | 0.036 |
| Anhedonia  *(loss of pleasure and/or interest in people, things or activities)* | 8 (7 - 9.25) | 55.0% | 7 (7 - 9) | 46.3% | 8 (7 - 8.25) | 64.3% | **0.040** | 8 (8 - 8) | 76.9% | 8 (7 - 8.5) | 50.0% | 0.398 |
| Avoidance of situations, people or activities because of body image | 8 (7 - 8) | 55.0% | 8 (7 - 9) | 52.6% | 8 (7 - 9) | 60.7% | 0.473 | 8 (8 - 9) | 76.9% | 7 (7 - 8.5) | 42.9% | 0.144 |
| **Items rated highly important by Allied Health practitioners only** | | | | | | | | | | | | |
| Digestive symptoms  *E.g. strong burping or belching, indigestion, urgent bowel movements, constipation* | 8 (7 - 8.75) | 65.0% | 8 (7 - 9) | 62.1% | 8 (7 - 9) | 67.9% | 0.517 | 8 (7 - 9) | 61.5% | 9 (7 - 9.25) | 71.4% | 0.252 |
| *^#^* Hormonal changes  *E.g. hormone imbalance, changes to menstrual cycle* | 7 (7 - 8.75) | 40.0% | 7 (5 - 8) | 33.7% | 8 (7 - 9) | 60.7% | **0.008** | 7 (6 - 9) | 46.2% | 8 (7 - 9.25) | 71.4% | 0.150 |
| Self-efficacy  *(belief in own ability to succeed)* | 8 (7 - 9.25) | 60.0% | 8 (7 - 9) | 55.8% | 8 (7 - 8.25) | 60.7% | 0.562 | 7.5 (7 - 8) | 46.2% | 8 (7.5 - 8.5) | 71.4% | 0.228 |

** Includes nurses, dieticians, psychologists, and researchers*

*^#^ Additional outcome suggested during Round 1 (received only one round of voting)*

*Results are presented as median (25^th^ – 75^th^ percentiles) and percentage of participants within each group. Significance indicates differences between groups (a) pre-surgical patients, post-surgical patients and healthcare practitioners, and (b) between bariatric practitioners and allied health practitioners.*


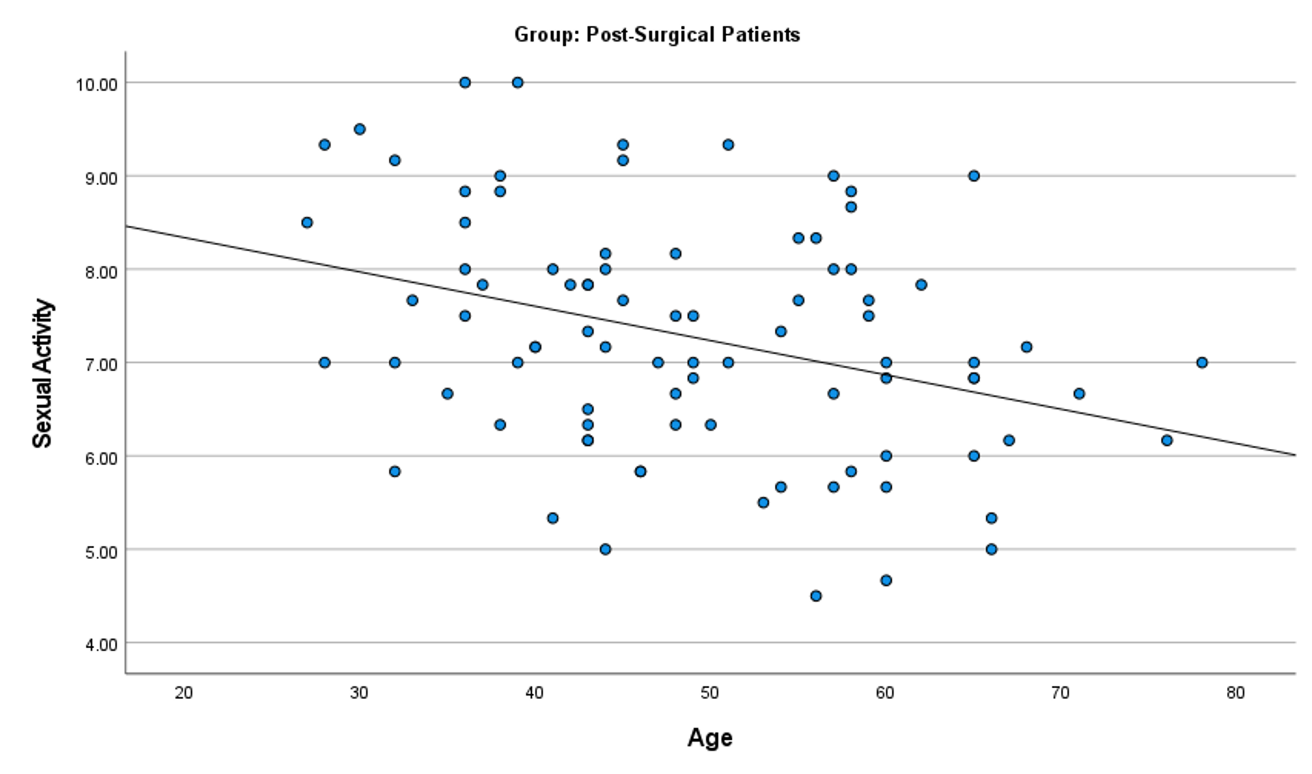
**Figure S1.** Correlation between mean importance score for the domain ‘Sex’ and participant age in the post-surgical patient group

**Figure S2:** Correlation between post-surgical patient time since surgery (months) and mean importance score for the domains (A) ‘Quality of Life’ and (B) ‘Sleep’


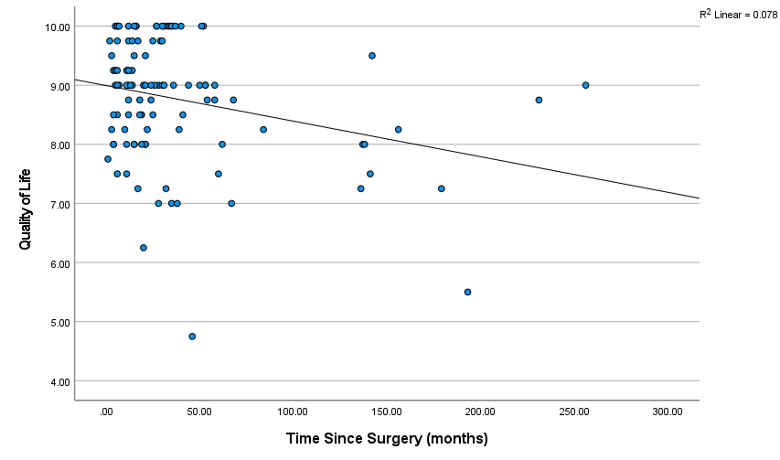

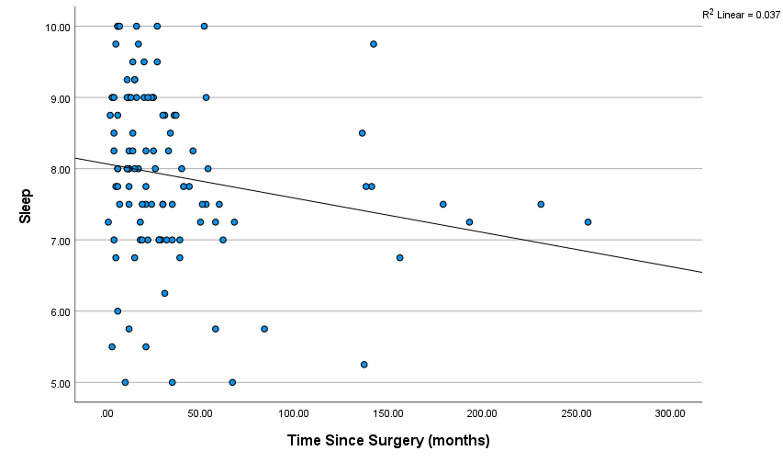


**(A)**

**(B)**

**General Quality of Life**
